# Relationship Between Optic Nerve Head Area and Peripapillary Retinal Nerve Fiber Layer Thickness in Laboratory Beagles

**DOI:** 10.1111/vop.70008

**Published:** 2025-02-24

**Authors:** Colleen E. Dwyer, Christine D. Harman, Mylee R. Haught, Frank R. Lawrence, Amanda L. Jacobson, Kristin L. Koehl, András M. Komáromy

**Affiliations:** ^1^ Department of Small Animal Clinical Sciences, College of Veterinary Medicine Michigan State University East Lansing Michigan USA; ^2^ Department of Small Animal Clinical Sciences, College of Veterinary Medicine University of Florida Gainesville Florida USA; ^3^ Center for Statistical Training and Consulting Michigan State University East Lansing Michigan USA

**Keywords:** canine, confocal scanning laser ophthalmoscopy, micropapilla, optic nerve axons, optical coherence tomography, retinal ganglion cells

## Abstract

**Objective:**

To determine if the area of the canine optic nerve head (ONH) is related to the peripapillary retinal nerve fiber layer (RNFL) thickness.

**Animals Studied:**

A total of 44 eyes of 22 adult normal laboratory Beagles.

**Procedures:**

In this retrospective study, confocal scanning laser ophthalmoscopy (cSLO) and spectral domain optical coherence tomography (SD‐OCT) images were evaluated. The sum of eight RNFL thickness measurements along the 12° circular OCT b‐scan around the ONH was used to represent the total number of retinal ganglion cell (RGC) axons. The area of the ONH was measured by tracing the outer edges of the ONH in the cSLO images and adjusted for axial globe length. Descriptive and Pearson correlation statistics were used to describe the distribution of the ONH sizes and the relationship between total RNFL thickness and ONH area.

**Results:**

The ONH area distribution for all eyes combined was approximately symmetric with an overall mean (± standard deviation) of 129 ± 35 degrees^2^. The ONH areas between right and left eyes showed a strong, significant linear correlation (*r* = 0.83, *p* = 0.00). The mean sum of the eight RNFL thicknesses measured along the 12° circular OCT b‐scan around the ONH was 448 ± 50 μm. There was a moderate, significant positive linear correlation between peripapillary RNFL thickness and ONH area (*r* = 0.43, *p* = 0.004).

**Conclusions:**

In laboratory Beagles, the ONH area is partly affected by the peripapillary RNFL thickness, representing the total RGC axon number forming the optic nerve.

## 1 Introduction

The morphologic and functional assessment of the optic nerve is an important part of the dog's ophthalmic and neurologic examination. The optic nerve mainly consists of the retinal ganglion cell (RGC) axons that connect the retina to the brain. There are an estimated 115 000–150 000 axons in the canine optic nerve [1, 2, 3, 4]. The normal canine optic nerve head (ONH) size is highly variable, as assessed by direct and indirect ophthalmoscopy, fundus photography, and confocal scanning laser ophthalmoscopy (cSLO) [1, 5]. In dogs, the size of the ONH is believed to be determined by the number of axons and the degree of myelination of the prelaminar ONH [1, 5]. The amount of myelin gradually increases in early life, with myelination of the optic nerve progressing from the brain to the eye during the first few weeks of life [6]. In addition to age, the degree of canine ONH myelination is also affected by the breed [5].

Congenitally small ONHs are categorized as either optic nerve hypoplasia or micropapilla. Optic nerve hypoplasia is clinically defined as a small ONH associated with severe congenital visual impairment or complete blindness, with severely reduced or negative dazzle and pupillary light reflexes [1, 7, 8, 9, 10, 11, 12]. In contrast, micropapilla is a small ONH that is not associated with any clinically detectable visual impairment and with normal dazzle and pupillary light reflexes [1, 8, 9]. To our knowledge, there is no quantitative definition of canine micropapilla, and the diagnosis tends to be a subjective assessment of small ONH size.

Based on the significant differences in the impact on eyesight, there are different breeding recommendations for optic nerve hypoplasia and micropapilla issued by the Genetics Committee of the American College of Veterinary Ophthalmologists (ACVO): The breeding of dogs with micropapilla is allowed (“breeder option”), but there is an unequivocal recommendation against the breeding of dogs affected by optic nerve hypoplasia [8]. Because of the potential genetic relationship between the two conditions [1, 9], the Hereditary Eye Diseases Committee of the European College of Veterinary Ophthalmologists (ECVO), on the other hand, recommends against the breeding of any dog diagnosed with either optic nerve hypoplasia or micropapilla [13].

While the lack of RGCs and their axons is well‐defined in optic nerve hypoplasia [10, 11, 12, 14], the underlying explanation for micropapilla is poorly understood: Is the abnormally small but functional ONH solely the result of poor pre‐laminar ONH myelination, or is there also a decrease in the number of axons compared to normal‐sized canine ONH [1, 6]? To our knowledge, no published studies address this question by correlating the ONH size with the number of RGC axons in dogs.

The purpose of this study was to test the hypothesis that the size of the canine ONH is related to the number of RGC axons forming the optic nerve. Using in vivo high‐resolution imaging, we examined a group of young adult laboratory Beagles with similar background genetics to compare the relationship between ONH size and peripapillary retinal nerve fiber layer (RNFL) thickness, the latter being determined by the total number of axons forming the optic nerve [15, 16].

## 2 Materials and Methods

### 2.1 Study Design

In this retrospective, single‐center study, high‐resolution ocular fundus images of young adult, purpose‐bred laboratory Beagles were analyzed. These baseline images were taken before the dogs were enrolled in specific studies. The area of the ONH was determined on cSLO images and compared to RNFL thickness, which was circumferentially measured around the ONH on spectral‐domain optical coherence tomography (SD‐OCT) images.

### 2.2 Animals

A total of 44 eyes of 22 adult normal laboratory Beagles, equally divided into females and males, were examined. The dogs were primarily young adults with a median age of 0.8 years (0.6–3.0 years) and were considered normal and healthy. The dogs were purchased from a commercial vendor (Marshall BioResources; *n* = 19) or bred in‐house (*n* = 3) and were used for breeding and/or as normal controls for unrelated prospective studies. All dogs were genotyped for the G661R *ADAMTS10* missense mutation and were determined to be unaffected by *ADAMTS10*‐open‐angle glaucoma [17].

Routine ophthalmic examination by a board‐certified veterinary ophthalmologist (AMK) using slit lamp biomicroscopy (Kowa SL17; Kowa Company) and indirect ophthalmoscopy (Keeler All Pupil II; Keeler Instruments and Double Aspheric Pan Retinal 2.2D; Volk Optical) did not reveal any significant ocular abnormalities that would have affected the optic nerve and the ability to image the ocular fundus. Menace responses, pupillary light, and dazzle reflexes were positive in all eyes enrolled in this study. Normal iridocorneal angle morphology was confirmed by gonioscopy using a RetCam II with a 130° lens (Clarity Medical Systems) following the application of ocular surface anesthesia (proparacaine HCl 0.5% ophthalmic solution; Alcon Laboratories Inc.) and viscoelastic coupling agent (OptixCare Plus Eye Lube). Routine fundus images were also obtained with the RetCam II and 130° lens for documentation. For all dogs, routine diurnal intraocular pressure (IOP) measurements by rebound tonometry (Icare TonoVet) were within normal limits (10–20 mmHg).

During the study, the dogs were group‐housed in the same environment at the Michigan State University College of Veterinary Medicine with a 12 h/12 h light/dark cycle and fed the same diet. The study complied with the Association for Research in Vision and Ophthalmology (ARVO) Statement for the Use of Animals in Ophthalmic and Vision Research and was approved by the Michigan State University Institutional Animal Care and Use Committee (IACUC).

### 2.3 Imaging

High‐resolution imaging with cSLO and OCT was performed under general anesthesia for the best possible image quality. Pupils were dilated with tropicamide 1% ophthalmic solution (Akorn Inc.). The dogs were premedicated with intravenous butorphanol tartrate (0.2–0.4 mg/kg; Bayer HealthCare LLC) and acepromazine maleate (0.02 mg/kg; PromAce; Boehringer Ingelheim Vetmedica Inc.), and general anesthesia was induced with intravenous propofol (2–4 mg/kg; PropoFlo 28; Zoetis Inc.) and midazolam HCl (0.2 mg/kg; Avet Pharmaceuticals Inc.). Following endotracheal intubation, general anesthesia was maintained with isoflurane/O_2_ gas anesthesia (Isothesia; Henry Schein).

Once the animals were under anesthesia, ocular surface anesthesia (proparacaine HCl 0.5% ophthalmic solution) was administered, and the eyes were positioned for imaging with conjunctival stay sutures (4–0 silk; Ethicon Inc.). The corneal surface was maintained with regular application of balanced salt solution (BSS; Alcon Laboratories Inc.). Imaging was performed with the Spectralis HRA + OCT combined cSLO and SD‐OCT system (Heidelberg Engineering) equipped with a 30° noncontact lens (Heidelberg Engineering). One hundred OCT b‐scans on a 12° circle centered on the ONH were averaged using Spectralis’ automatic real‐time function. Following OCT imaging, axial lengths were measured by A‐scan ultrasound biometry (A‐scan; Scanmate, DGH Technology).

### 2.4 Image Analysis

**FIGURE 1:**
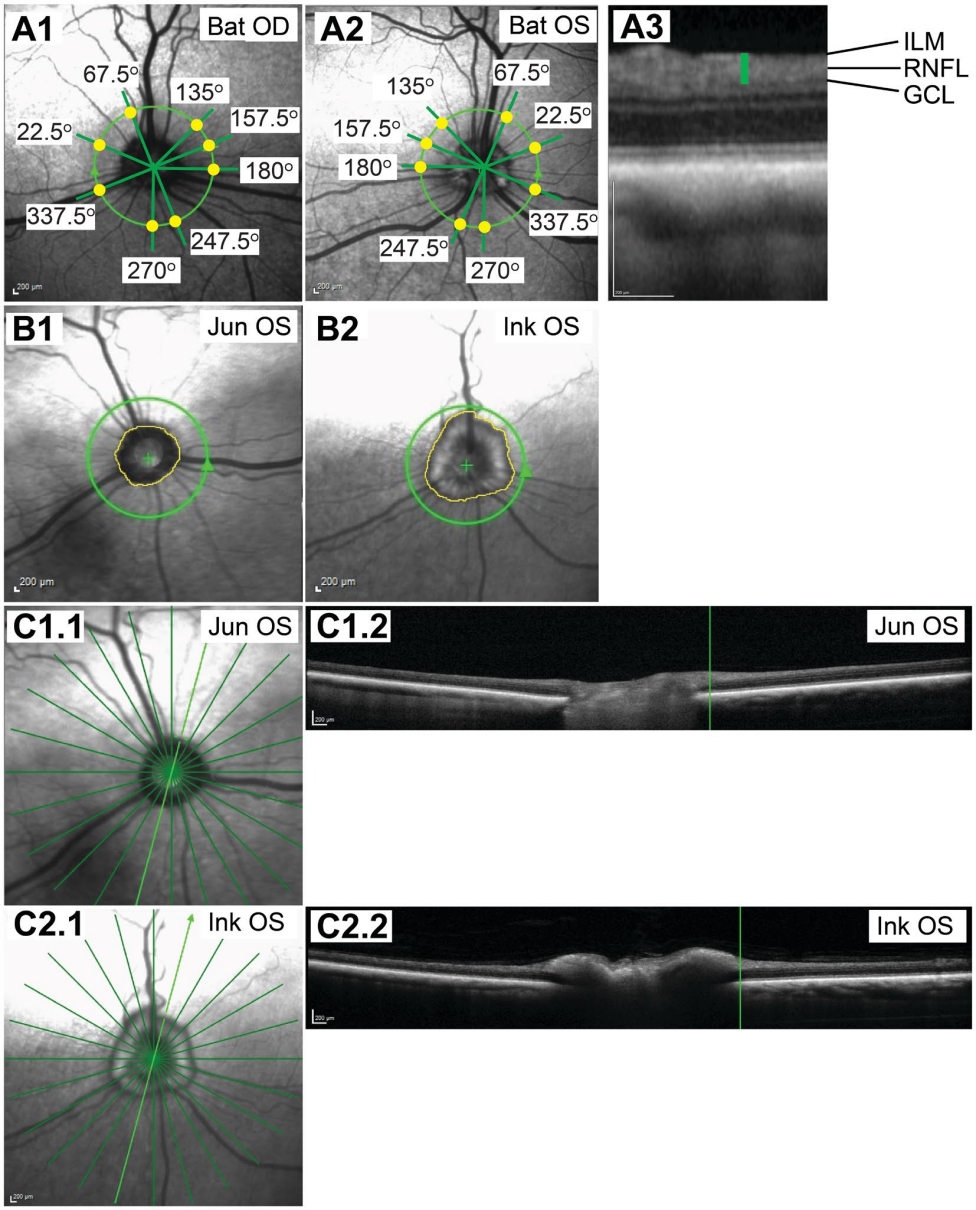
Measurements of peripapillary RNFL thickness and ONH area. The yellow dots in panels (A1) and (A2) show where RNFL thickness was measured on the 12° circular OCT b‐scans around the ONH on OD and OS, respectively. The RNFL thickness was measured from the inner border of the ganglion cell layer (GCL) to the inner limiting membrane (ILM) (A3). The ONH areas were measured by tracing the outer edge on cSLO images in ImageJ (B1 and B2, yellow line). Two different eyes are shown as examples (B1 and B2). Panels (C1.1) and (C2.1) show the same ONHs as in (B1) and (B2) but with the location of the 30° stars, that is, the location of 12 OCT b‐scans through the ONH. One of these b‐scans (bright green lines in C1.1 and C2.1) is shown for each eye in panels (C1.2) and (C2.2) to visualize the edge of the ONH (vertical green line). The labels in the upper right corners of the images show the dog ID and the eye (OD or OS). Calibration bars = 200 μm. cSLO, Scanning Laser Ophthalmoscopy; GCL, Ganglion Cell Layer; ILM, Inner Limiting Membrane; OCT, Optical Coherence Tomography; OD, Right Eye; ONH, Optic Nerve Head; OS, Left Eye; RNFL, Retinal Nerve Fiber Layer.

Using the Heidelberg Eye Explorer software version 1.10.12.0 (Heidelberg Engineering), the RNFL thickness was measured manually in eight locations, two per quadrant (superior nasal, superior temporal, inferior nasal, inferior temporal) on the 12° circular OCT b‐scan around the ONH (Figure 1A): 22.5°, 67.5°, 135°, 157.5°, 180°, 247.5°, 270°, and 337.5°. These locations were selected to avoid retinal blood vessels in most canine eyes [18]. If, in a particular eye, one of these locations overlapped with a retinal blood vessel, we moved to an adjacent region along the circular OCT b‐scan to perform the RNFL thickness measurement. The sum of all eight peripapillary RNFL thickness measurements was used to represent the total RGC axon number in the statistical analyses.

The area of the ONH was measured by importing the cSLO images into ImageJ [19] and tracing the outer edges of the ONH (Figure 1B). The 200‐μm calibration bar was used as a reference. Using the axial length from the A‐scan and the retinal magnification factor, the measured ONH area (in mm^2^) was converted to an angular area (in degrees^2^) in a schematic eye to account for global size differences [20, 21, 22, 23]. Figure 1C shows OCT b‐scans through two ONHs to visualize the transition from myelination to retinal layering. The 12° circular OCT b‐scan was located outside the myelinated ONH in all enrolled eyes.

### 2.5 Statistical Analysis

The distribution of ONH areas was analyzed using descriptive statistics (Microsoft Excel). Linear Pearson correlations between the right (OD) versus left (OS) eye ONH areas and between RNFL thickness and ONH area were calculated in R v4.3.0 [24].

## 3 Results

### 3.1 Distribution of ONH Areas

**FIGURE 2:**
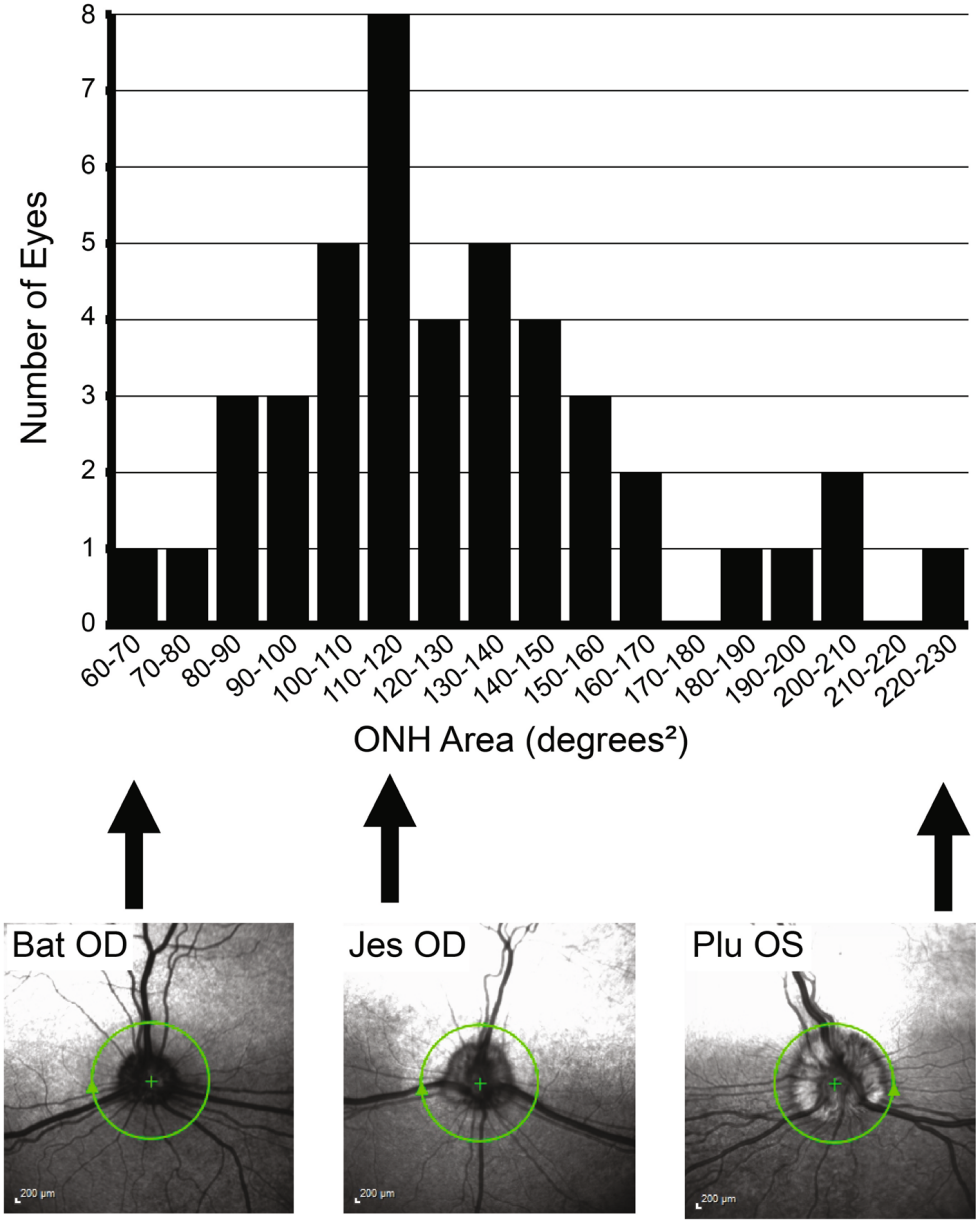
Distribution of ONH areas. The bar chart at the top shows the distribution of ONH areas of all the Beagle eyes included in the study. The three cSLO images at the bottom show representative eyes for the categories 60–70, 110–120, and 220–230 degrees^2^. The standard‐sized 12° green circles indicate the location of the OCT b‐scans around the ONH. The cSLO images are labeled with the dog ID and eye. Calibration bars = 200 μm. cSLO, Confocal Scanning Laser Ophthalmoscopy; OD, Right Eye; ONH, Optic Nerve Head; OS, Left Eye.

Optic nerve head area distribution was approximately symmetric with an overall mean (± standard deviation) of 129 ± 35 degrees^2^ (Figure 2). The median ONH area was 121 degrees^2^ with a minimum of 67 degrees^2^ and a maximum of 221 degrees^2^ [2]. Figure 2 includes representative cSLO images showing the range of ONH areas in the Beagle population studied. While we considered the smallest ONH areas observed in our population of Beagles to be consistent with micropapilla, we did not assign this diagnosis to our ONH area categories due to the lack of a clear definition. The continuous transition from small to larger ONH areas would have rendered a specific threshold between normal and abnormal ONH size arbitrary in our study population.

**FIGURE 3:**
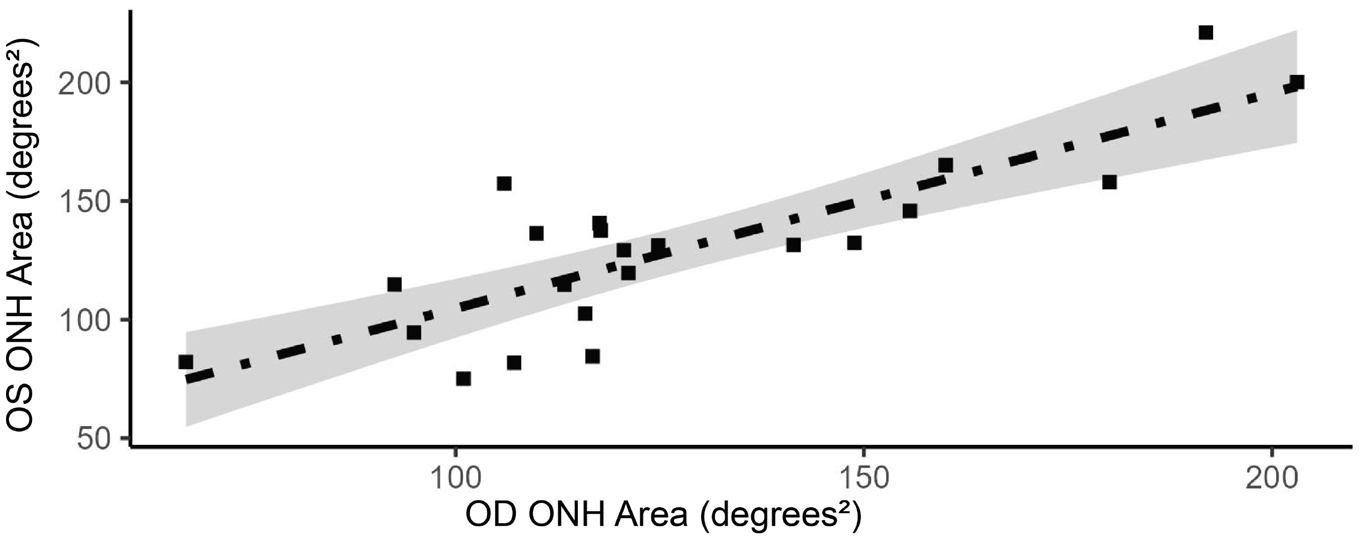
OD‐OS ONH area comparison. There was a strong, significant positive linear correlation between OD and OS ONH areas (*r* = 0.83; *p* = 0.00). The shaded area represents the 95% local confidence interval. OD, Right Eye; ONH, Optic Nerve Head; OS, Left Eye (OS).

There was a strong, significant positive linear correlation between OD and OS ONH areas (*r* = 0.83, *p* = 0.00; Figure 3). Only 12 of 44 eyes were not within the 95% local confidence interval. While 5 of the 22 dogs showed the same ONH area size category for the 2 eyes, the 2 ONH areas were in different size categories for the other 17 dogs (Figure 2). Based on this observation, we did not combine the data of OD and OS in our comparisons of peripapillary RNFL thickness and ONH area, but evaluated each eye separately.

### 3.2 Relationship Between Peripapillary RNFL Thickness and ONH Area

**FIGURE 4:**
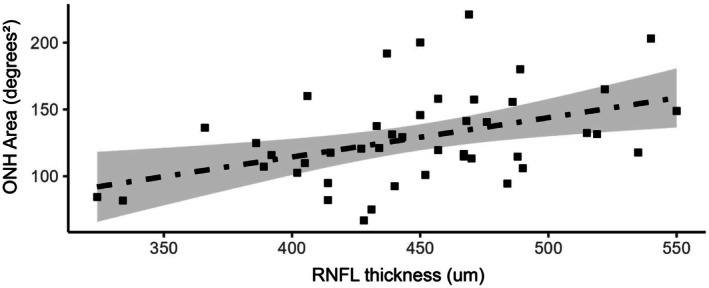
Peripapillary RNFL thickness vs. ONH area comparison. There was a moderate, significant positive linear correlation between peripapillary RNFL thickness and ONH area (*r* = 0.43; *p* = 0.004). The shaded area represents the 95% local confidence interval. ONH, Optic Nerve Head; RNFL, Retinal Nerve Fiber Layer.

The main purpose of our study was the comparison of peripapillary RNFL thickness with ONH area. The mean sum of the eight RNFL thicknesses measured along the 12° circular OCT b‐scan around the ONH was 448 ± 50 μm (Figure 1). The median peripapillary RNFL thickness sum was 450 μm with a minimum of 324 μm and a maximum of 550 μm. We found a moderate, significant positive linear correlation between ONH area and peripapillary RNFL thickness (*r* = 0.43, *p* = 0.004; Figure 4), with 17 of 44 eyes within the 95% local confidence interval. This supports our hypothesis that the canine ONH area is at least in part affected by the total number of RGC axons.

## 4 Discussion

The results of our study suggest that the laboratory Beagle ONH area is at least in part related to the peripapillary RNFL thickness. We can further conclude that the size of the ONH is not solely determined by the degree of myelination. Even though the amount of prelaminar myelin also affects the size of the ONH, and in fact obscures the true size of the ONH [1, 5], the conclusions of our RNFL versus ONH area comparisons are valid because retinal thickness measurements along the 12° circular b‐scan occurred outside of the myelinated ONH. The distance of the standardized circular b‐scan from the ONH edge varied depending on the size and shape of the ONH. The significant linear correlation between RNFL thickness and ONH area supports our hypothesis that the laboratory Beagle ONH area is affected by the number of RGC axons.

The general notion, based on studies in other species, that the size of the ONH is not only related to the degree of myelination but, more importantly, to the number of RGC axons, has never been firmly documented in dogs to the best of our knowledge [1, 3, 4, 25, 26]. Bemis et al. showed in 2017 a moderate correlation (*r* = 0.48, *p* < 0.01) between RNFL thickness and ONH area in Beagles, but the sample size was smaller (*n* = 12 dogs) with a much narrower distribution of ONH sizes compared to our current study [27]. Furthermore, the ONH size was not adjusted for globe size, which may not be critical in a population of uniform laboratory Beagles [27]. The main focus of that study was the reproducibility and repeatability of OCT imaging of the ONH in normal Beagle eyes [27]. We believe the current study may be the most extensive canine study providing data supporting the strong relationship between total RGC axon number forming the optic nerve (as estimated by peripapillary RNFL thickness [15, 16]) and ONH area.

Small ONHs without vision loss are referred to as micropapilla [1]. Unfortunately, there is no quantitative definition for micropapilla in dogs, and the diagnosis tends to be a subjective assessment by the clinician. We consider the two smallest ONHs in our study (67 and 75 degrees^2^; Figure 2) to be consistent with micropapilla. These two ONHs had RNFL thicknesses closer to the average (448 μm), 428 and 431 μm, respectively, outside the 95% confidence interval (Figure 4). On the other hand, the two eyes with the thinnest peripapillary RNFL (324 and 334 μm) also had very small ONHs, possibly consistent with micropapilla, and were located within the 95% confidence interval (84 and 82 degrees^2^; Figure 4).

At least for the laboratory Beagle, our data contradict the notion that a small ONH is associated with a normal‐sized optic nerve and that the smaller size of the ONH is solely the effect of less myelin [28]. Our data also support the notion that micropapilla and optic nerve hypoplasia could be related, with micropapilla being a less severe form of optic nerve hypoplasia but with both conditions being caused by a decreased number of RGC axons compared to normal‐sized ONHs [1, 9, 13]. This supports the ECVO's recommendation against the breeding of dogs with micropapilla [13]. However, our group of laboratory Beagles shows that there is a gradual transition from micropapilla to normal‐sized ONHs and that the diagnosis in a particular dog can be quite subjective. This observation of possible ambiguity with the diagnosis of micropapilla supports the ACVO's breeding recommendation of a “breeder option” for this condition [8].

A major strength of our study was the focus on a group of laboratory Beagles with similar background genetics and raised under similar environmental conditions. Our study population was well balanced regarding the sexes.

Our study had several important limitations. The study was limited to a relatively small group of laboratory Beagles, and future studies will have to confirm if our conclusions apply to the general dog population. For example, it will be interesting to compare peripapillary RNFL thicknesses across breeds with different extents of ONH myelination [5].

Another limitation of our study was that the total optic nerve axon numbers could not be assessed in this in vivo study and used the peripapillary RNFL thickness as an indirect measure for all the RGC axons converging at the ONH and forming the optic nerve. This method allowed the non‐invasive in vivo examination of more dogs with similar background genetics. The gold standard of axon counts or estimates on histologic optic nerve cross‐sections would have been more accurate but would have required the enucleation or sacrifice of numerous normal, healthy dogs [3, 26]. This was not a feasible and ethical option.

Measurement of the RNFL along the 12° circular OCT b‐scan appeared to be the most consistent method for comparing eyes from different dogs. Because of myelination, it is generally impossible to identify the actual edge of the canine ONH and a more consistent location for RNFL thickness measurement than the 12° circular scan.

While optic nerve hypoplasia is associated with severe functional deficits, no such data exist for micropapilla. The small number of RGC axons with micropapilla suggested in our study indicates that a functional deficit, such as decreased visual acuity, may be present, albeit very difficult to measure, and therefore not clinically significant in affected dogs. While functional testing was not part of this study, we ensured, with our baseline ophthalmic examinations, that all enrolled dogs and eyes had positive menace responses, dazzle, and pupillary light reflexes.

In conclusion, our study provides the strongest support to date that the size of the canine ONH area is significantly correlated with the number of RGC axons and is not only determined by the degree of myelination.

## Author Contributions

**Colleen E. Dwyer:** conceptualization, data curation, formal analysis, investigation, methodology, visualization, writing – original draft, writing – review and editing. **Christine D. Harman:** conceptualization, data curation, investigation, methodology, project administration, supervision, writing – review and editing. **Mylee R. Haught:** data curation, formal analysis, investigation, methodology, validation, visualization. **Frank R. Lawrence:** formal analysis, writing – review and editing. **Amanda L. Jacobson:** investigation, methodology, writing – review and editing. **Kristin L. Koehl:** conceptualization, investigation, methodology, writing – review and editing. **András M. Komáromy:** conceptualization, data curation, formal analysis, funding acquisition, investigation, methodology, project administration, resources, supervision, visualization, writing – original draft, writing – review and editing.

## Ethics Statement

This study adhered to the Association for Research in Vision and Ophthalmology (ARVO) Statement for the Use of Animals in Ophthalmic and Vision Research and was approved by the Michigan State University Institutional Animal Care and Use Committee (IACUC).

## Conflicts of Interest

AM Komáromy is a consultant for Reichert Technologies and W. L. Gore & Associates Inc. AM Komáromy received research funding from PolyActiva Pty. Ltd. and CRISPR Therapeutics while the presented work was conducted. While AM Komáromy also serves as Editor‐in‐Chief of Veterinary Ophthalmology, he was not involved in the review of this manuscript. All other authors declare no conflicts of interest.

## Data Availability

The data that support the findings of this study are available from the corresponding author upon reasonable request.
